# Rhinovirus and dsRNA Induce RIG-I-Like Receptors and Expression of Interferon β and λ_1_ in Human Bronchial Smooth Muscle Cells

**DOI:** 10.1371/journal.pone.0062718

**Published:** 2013-04-29

**Authors:** Jenny Calvén, Yuliana Yudina, Lena Uller

**Affiliations:** Unit of Respiratory Immunopharmacology, Department of Experimental Medical Science, Lund University, Lund, Sweden; University of Tennessee Health Science Center, United States of America

## Abstract

Rhinovirus (RV) infections cause exacerbations and development of severe asthma highlighting the importance of antiviral interferon (IFN) defence by airway cells. Little is known about bronchial smooth muscle cell (BSMC) production of IFNs and whether BSMCs have dsRNA-sensing receptors besides TLR3. dsRNA is a rhinoviral replication intermediate and necrotic cell effect mimic that mediates innate immune responses in bronchial epithelial cells. We have explored dsRNA-evoked IFN-β and IFN-λ_1_ production in human BSMCs and potential involvement of TLR3 and RIG-I-like receptors (RLRs). Primary BSMCs were stimulated with 0.1–10 µg/ml dsRNA, 0.1–1 µg/ml dsRNA in complex with the transfection agent LyoVec (dsRNA/LyoVec; selectively activating cytosolic RLRs) or infected with 0.05–0.5 MOI RV1B. Both dsRNA stimuli evoked early (3 h), concentration-dependent IFN-β and IFN-λ_1_ mRNA expression, which with dsRNA/LyoVec was much greater, and with dsRNA was much less, after 24 h. The effects were inhibited by dexamethasone. Further, dsRNA and dsRNA/LyoVec concentration-dependently upregulated RIG-I and MDA5 mRNA and protein. dsRNA and particularly dsRNA/LyoVec caused IFN-β and IFN-λ_1_ protein production (24 h). dsRNA- but not dsRNA/LyoVec-induced IFN expression was partly inhibited by chloroquine that suppresses endosomal TLR3 activation. RV1B dose-dependently increased BSMC expression of RIG-I, MDA5, IFN-β, and IFN-λ_1_ mRNA. We suggest that BSMCs express functional RLRs and that both RLRs and TLR3 are involved in viral stimulus-induced BSMC expression of IFN-β and IFN-λ_1_.

## Introduction

Respiratory viral infections, human rhinoviruses (RV) in particular, are considered major triggers of severe asthma exacerbations in children and adults [1], [2]. The airway epithelium is the primary target for RV and the main site for viral replication. However, *in situ* hybridisation analyses of bronchial biopsies from infected subjects have demonstrated presence of RV in the subepithelial mucosa layer [3] suggesting that additional bronchial wall cells could be targets for viral infection. During RV replication biologically active double-stranded RNA (dsRNA) molecules are produced. In bronchial epithelial cells dsRNA is known to produce innate immune effects through specific pattern recognition receptors (PRRs) including Toll-like receptor 3 (TLR3) and the cytosolic RNA helicases/RIG-I-like receptors (RLRs), retinoic acid inducible gene-I (RIG-I) and melanoma differentiation associated gene 5 (MDA5) [4], [5]. Activation of TLR3 and RLRs by dsRNA in turn activates transcription factors NF-κB and interferon regulatory factor 3 (IRF3) leading to upregulated gene expression and production of a variety of potent cytokines including proinflammatory TNF-α and CXCL8/IL-8 and antiviral interferons (IFNs) [4], [6], [7]. Severe asthma conditions are associated with necrotic cells and injury-repair processes in the bronchi [8], [9]. This aspect is important because molecules emanating from necrotic cells also activate PRRs producing effects that in part can be mimicked by dsRNA [10], [11], [12], [13].

Epithelial generation of type I and type III IFNs, such as IFN-β and IFN-λs, constitute a major host defence against respiratory viral infections [14], [15]. Other groups and we have previously shown that bronchial epithelial cells from asthmatic individuals exhibit deficient production of IFN-β and IFN-λs in response to RV infection and dsRNA [14], [16], [17]. Although these findings may not apply to all asthmatic cohorts [18] they suggest the possibility that deficient IFN production makes patients with asthma more susceptible to RV infection. Possibly contributing to desired effects in asthma, IFNs produce effects that may counteract development of Th2 inflammation [19], [20]. However, IFNs also have capacities to increase bronchial injury and inflammation [21], [22]. In a current study IFN-λ_1_ (IL-29) was strongly and exclusively associated with RV-induced exacerbations of asthma in children suggesting that IFN-λ_1_ could have a pathogenic role in RV-induced exacerbations [21], [23]. In accord with previous findings in adult asthma [14] the investigators further showed that IFN-λ_1_ production by airway epithelial cells obtained from children with asthma post-RV infection was suppressed compared with healthy epithelial cells [21]. Hence, it is not known which cells in the asthmatic airway may overproduce this type III IFN [23].

Traditionally, the interest in bronchial smooth muscle in airway diseases has concerned its contractile properties leading to airway obstruction. Although this interest remains with still unmet medical needs [24], during the last two decades the research focus has increasingly been on the synthetic and immunomodulatory properties of the bronchial smooth muscle cells (BSMCs) [25]. BSMCs thus express functional TLRs with preferential expression of TLR3 [26]. Activation of TLR3 results in BSMC synthesis of a wide range of inflammatory cytokines and chemokines including CXCL8, IL-6, CCL5/RANTES and CCL11/eotaxin-1 [26], [27], [28], [29]. *In vitro* studies using primary BSMCs from donors with asthma have further shown that RV induces greater or equal amounts of IL-6 and CXCL8 in these diseased cells compared to cells from healthy donors [27]. However, data indicating that RV and dsRNA induce IFNs in healthy or diseased BSMCs have not been reported.

The aim of the current study was to investigate if primary human BSMCs could produce IFN-β and IFN-λ_1_ in response to rhinoviral stimuli with emphasis on synthetic dsRNA stimulation. We also explored expression of cytosolic RLRs, RIG-I and MDA5, in BSMCs and their possible functional importance for IFN production. Here we show for the first time that type I and type III IFNs are significantly induced by BSMCs exposed to RV and dsRNA and that cytosolic RLRs together with TLR3 have a role in the present IFN generation.

## Materials and Methods

### Human BSMC Culture

Primary human BSMCs from three healthy donors were purchased from Lonza (Walkersville, MD, USA) as cryopreserved cells. Cells were cultured in Smooth Muscle Growth Medium (SmGM-2; Clonetics, San Diego, CA, USA) in 5% CO_2_ and 37°C. At confluence, cells exhibited a hill and valley shaped growth pattern. For experiments, BSMCs were seeded into 6- or 12-well plates (Nunc, Life Technologies, Carlsbad, CA, USA) and when 80–90% confluent the growth medium was replaced with SmGM-2 containing reduced FBS (0.5%) for 24 h prior to all experiments.

### Stimulation of Cells with dsRNA and dsRNA/LyoVec

For all experiments cells were used at passage 4–8. BSMCs were stimulated with either the dsRNA analogue and TLR3 ligand, polyinosine-polycytidylic acid (Poly (I:C); InvivoGen San Diego, CA, USA) or the RIG-I-like receptor ligand Poly(I:C)/LyoVec (the dsRNA analogue in complex with the transfection agent LyoVec; InvivoGen). Unstimulated cells were kept as controls. The substances were tested in the range 0.1–10 µg/ml and 0.1–1 µg/ml, respectively, to establish concentration-dependent effects. When indicated, 10 µg/ml chloroquine (Sigma-Aldrich, Gillingham, UK) an agent that inhibits endosomal acidification [30], or 1 µg/ml dexamethasone (Sigma-Aldrich) were added 1 h prior to stimulation with dsRNA or dsRNA/LV and present until the end of the experiments. After 3 or 24 h stimulation, the cell supernatants were removed and collected for protein analysis and cells lysed and harvested for either mRNA or western blot analyses as described below.

### Infection with Rhinovirus 1B

A series of separate experiments were performed in which BSMCs were infected with RV1B grown in Ohio HeLa cells (European Collection of Cell Cultures) as previously described [31]. RV1B in different levels of multiplicity of infection (MOI), 0.05–0.5 MOI, were used to infect BSMCs for 1 h at room temperature with shaking. Virus preparations were then removed, cells washed 3 times in 1 ml PBS, and 1 ml fresh medium (without antibiotics) was added prior to incubation in 37°C and 5% CO_2_. When indicated, 10 µg/ml chloroquine was added simultaneously to RV1B infection and again after RV1B removal to be present until the end of the experiment. As controls, cells without virus exposure underwent the same infection procedure (with the absence of RV) as RV-infected cells. All cells were lysed and harvested for mRNA analysis 24 or 48 h post-infection.

### RNA Extraction and Quantification of Gene Expression by RT-qPCR

Total RNA was extracted from primary BSMCs using the RNA extraction kit NucleoSpin RNA II (Machery-Nagel, Düren, Germany), according to the manufacturer’s instructions. An RT-kit (Primerdesign, Southampton, UK) was used to reverse transcribe 1 µg of total RNA to cDNA and qPCR was performed as previously described [17]. In brief, an iCycler iQ real-time detection system (Stratagene, Mx3000P, La Jolla, CA, USA) with standard cycling parameters was used to perform thermocycling and real-time detection of PCR products. The following primer sequences were used for IFN-β, IFN-λ_1_, RIG-I, MDA5, TNF-α, CXCL8 and RV1B. IFN-β: TTACTTCATTAACAGACTTACAGGT (forward) and TACATTAGCCATCAGTCACTTAAAC (reverse), IFN-λ_1_: ATGGGAACCTGTGTCTGAGAA (forward) and GGGTGAGAGGAAATAAATTAAGGAA (reverse), RIG-I: TTCTCTTGATGCGTCAGTGATA (forward) and CCGTGATTCCACTTTCCTGAA (reverse), MDA5: GTCTCGTCACCAATGAAATAGC (forward) and TTATACATCATCTTCTCTCGGAAATC (reverse), TNF-α: AGGTTCTCTTCCTCTCACATAC (forward) and ATCATGCTTTCAGTGCTCATG (reverse), CXCL8: CAGAGACAGCAGAGCACAC (forward) and AGCTTGGAAGTCATGTTTACAC (reverse), and RV1B: TGGGTGTTGTACTCTGTTATTCC (forward) and TTGCCTACTATTGGTCTTGTGTT (reverse). Genes of interest were normalised to the geometric means of two reference genes, ubiquitin c (UBC) and glyceraldehyde 3-phosphate dehydrogenase (GAPDH), using the ΔCt method. Within-group comparisons were normalized to one control sample, or in the case of RV1B to 0.1 MOI at 24 h, of a single experiment using the ΔΔCt method.

### ELISA Analysis of Cytokine Release in Cell Supernatants

Release of IFN-β (PBL InterferonSource, NJ, USA), IFN-λ_1_, CXCL8 and TNF-α (R&D Systems, Abingdon, UK) into BSMC supernatants was measured after 24 h treatment with dsRNA or dsRNA/LyoVec using ELISA kits according to the manufacturer’s instructions.

### Western Blot Analysis of Receptor Expression

RIG-I and MDA5 protein expression was quantified by western blot. Experiments were performed as described above, and after stimulation with dsRNA (24 h), dsRNA/LyoVec (24 h) or RV1B infection (24 or 48 h) cells were lysed using a sample buffer for western blot containing 250 nM HCL, 6% SDS, 100 mM DTT, 10% glycerol and 25 mM NaF. Coomassie blue was used to determine protein content for each sample and equal amount of each sample was then loaded and electrophoresed on a 10% polyacrylamide gel. The fractionated protein was blotted onto a PVDF membrane. After blocking in 5% non-fat milk dissolved in PBS-T, the membrane was incubated overnight with the following primary antibodies: rabbit polyclonal RIG-I antibody (Cell Signaling Technologies, Danvers, CA, USA; diluted 1∶1000), goat polyclonal MDA5 antibody (Santa Cruz Biotechnology Inc, CA, USA; diluted 1∶500) or mouse polyclonal GAPDH antibody used as an internal loading control (Cell Signaling Technologies; diluted 1∶1000). The following day, the membrane was washed and incubated with corresponding horse-radish peroxidase-conjugated secondary antibodies: anti-rabbit IgG (Fisher Scientific AB, Gothenburg, Sweden; diluted 1∶1000), anti-goat IgG (R&D Systems, diluted 1∶500) or anti-mouse IgG antibody (Cell Signaling Technologies; diluted 1∶10000). The immunoreactive proteins were detected with a chemiluminescent system (ECL kit, GE healthcare, Buckinghamshire, UK).

### Statistical Analysis

All data were analysed using the software GraphPad Prism version 5.0 (GraphPad Software, San Diego, CA, USA) and expressed as mean value with SEM. Significant variations between paired groups were determined by the non-parametric tests Friedman’s one-way ANOVA and Wilcoxon matched-pairs signed ranks test. P-values of less than 0.05 were considered statistically significant.

## Results

### Effect of dsRNA on BSMC Generation of the Antiviral Cytokines IFN-β and IFN-λ_1_

**Figure 1:**
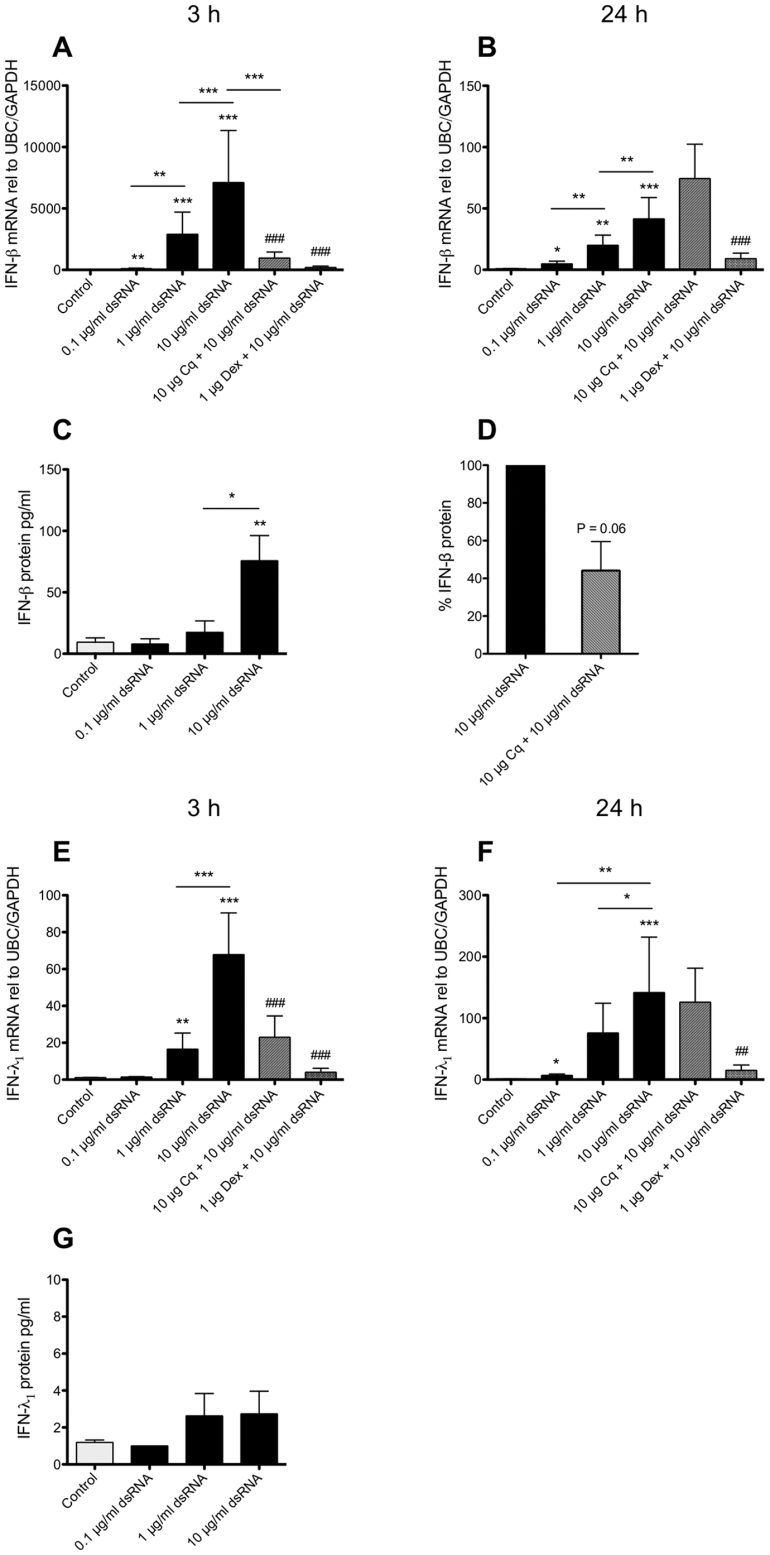
Effect of dsRNA on BSMC expression and production of IFN-β and IFN-λ_1_. BSMCs were stimulated with dsRNA and expression of IFN-β (A, B) and IFN-λ_1_ (E, F) mRNA was analysed by RT-qPCR after 3 h (A, E) or 24 h (B, F). Generation of IFN-β (C) and IFN-λ_1_ protein (G) was determined after 24 h in the cell supernatants by ELISA. Chloroquine (Cq) and dexamethasone (Dex) added 1 h prior to dsRNA stimulation inhibited the early (3 h) response, but only dexamethasone inhibited the late (24 h) mRNA response. IFN-β protein was reduced with chloroquine treatment (D). Data are presented as mean with SEM and n = 8–10 (BSMCs from three individual donors). *p≤0.05, **p≤0.01 and ***p≤0.001 compared to non-stimulated cells (control). ^##^p≤0.01 and ^###^p≤0.001 compared to 10 µg/ml dsRNA.

To date the possibility that BSMCs may be a source of type I and type III IFNs in viral-infected airways has not been reported. However, we now demonstrate that dsRNA produced an early (3 h) concentration-dependent induction of both IFN-β (7000-fold) and IFN-λ_1_ (65-fold) mRNA expression in BSMCs (Figure 1A and 1E). After 24 h, IFN-β expression was still increased but much less so (Figure 1B), whereas IFN-λ_1_ expression was more increased than at 3 h (Figure 1F). Chloroquine treatment, that is known to prevent endosomal acidification and thus antagonize TLR3 activation [30], reduced dsRNA-induced effects on IFN-β and IFN-λ_1_ expression at 3 h (Figure 1A and 1E) but was without inhibitory effect at 24 h (Figure 1B and 1F). Dexamethasone largely inhibited effects of dsRNA on IFN-β and IFN-λ_1_ at both 3 and 24 h (Figure 1A, 1B, 1E and 1F). To determine if the increased mRNA expression was associated with production of IFN protein, cell supernatants were analysed after 24 h. The BSMCs produced significant IFN-β protein in response to 10 µg/ml dsRNA (Figure 1C) with a 50% reduction by chloroquine (Figure 1D). IFN-λ_1_ protein only tended to increase in response to 1 and 10 µg/ml dsRNA (Figure 1G).

### RLR Activation Evokes Concentration-dependent BSMC Production of IFN-β and IFN-λ_1_

**Figure 2:**
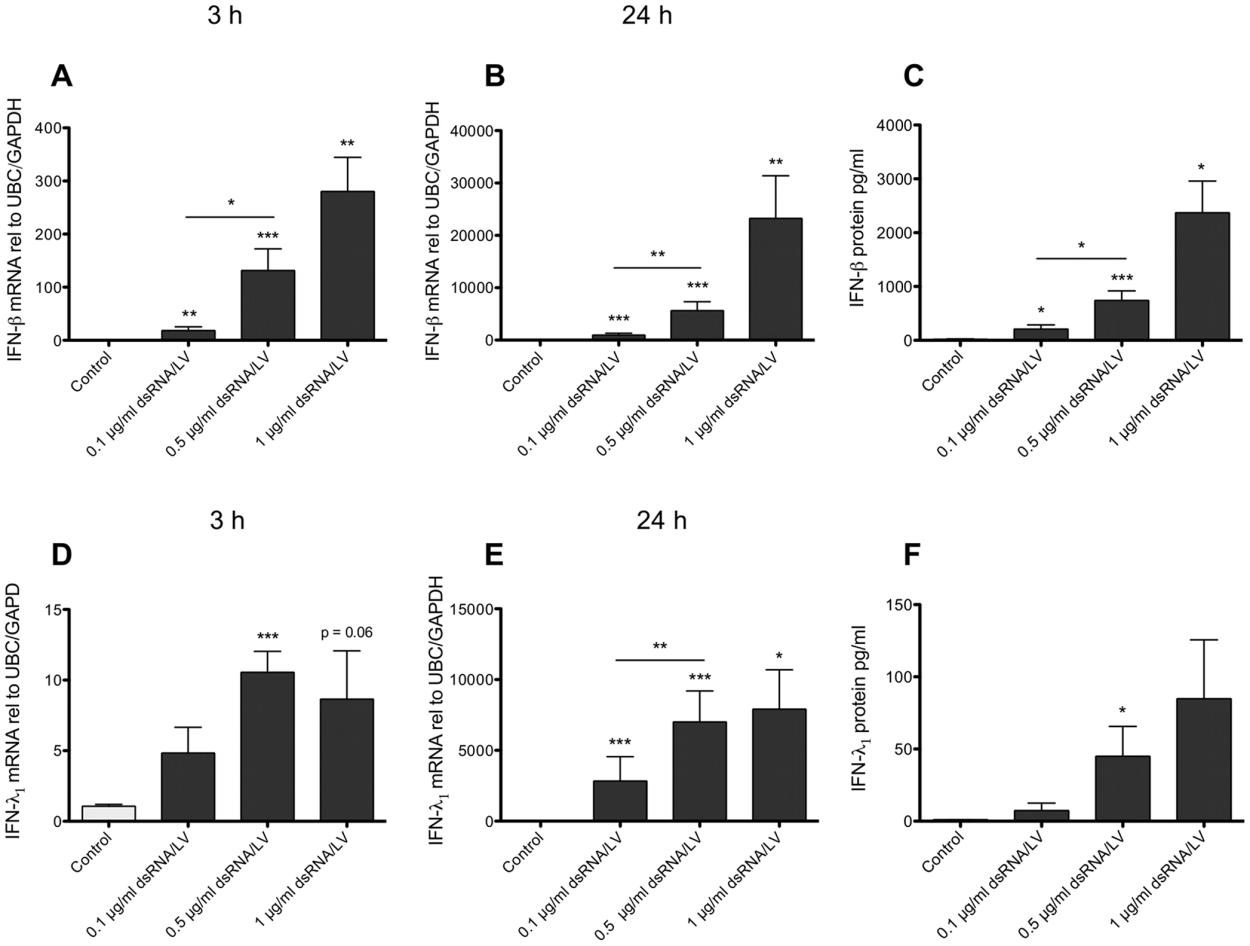
RLR activation by dsRNA/LyoVec evokes BSMC mRNA expression and protein production of IFN-β and IFN-λ_1_. IFN-β and IFN-λ_1_ mRNA expression was increased by dsRNA/LyoVec (dsRNA/LV) after 3 h (A, D) and 24 h (B, E) and so was secretion of IFN-β and IFN-λ_1_ after 24 h (C, F). Data are presented as mean with SEM and n = 8–10 (BSMCs from three individual donors). *p≤0.05, **p≤0.01 and ***p≤0.001 compared to non-stimulated cells (control).

The limited inhibitory effect of chloroquine at 24 h (see above) suggested that TLR3 was not the only PRR involved in dsRNA-induced IFNs. To address the possibility of a role of RLRs we examined effects of treatment with dsRNA/LyoVec, a dsRNA construct that confines dsRNA effects to the cytosol and thus selectively activates the RLRs [32], [33]. dsRNA/LyoVec concentration-dependently induced IFN-β (300-fold) and IFN-λ_1_ (10-fold) expression after 3 h treatment (Figure 2A and 2D). By 24 h the expression of IFN-β (25000-fold) and IFN-λ_1_ (8000-fold) was markedly enhanced by dsRNA/LyoVec (Figure 2B and 2E). Treatment with LyoVec alone or in complex with an unspecific RNA sequence had no effect on IFN expression (data not shown). We then used chloroquine treatment to examine whether dsRNA/LyoVec produced effects by stimulation of TLR3. Supporting the specificity of dsRNA/LyoVec for RLRs, chloroquine (10 µg/ml) did not reduce IFN-β or IFN-λ_1_ mRNA at 3 and 24 h (Figure S1). The effect of dsRNA/LyoVec on IFN mRNA expression also caused IFN protein production as demonstrated by increased concentrations of IFN-β and IFN-λ_1_ protein in the BSMC culture medium (Figure 2C and 2F). This effect was particularly prominent with respect to IFN-β. The magnitude of the IFN-β and IFN-λ_1_ responses to dsRNA/LyoVec was also much greater than observed after dsRNA treatment (Figure 1C and 1G).

### dsRNA Induces Upregulation of RLR Expression in BSMCs

**Figure 3:**
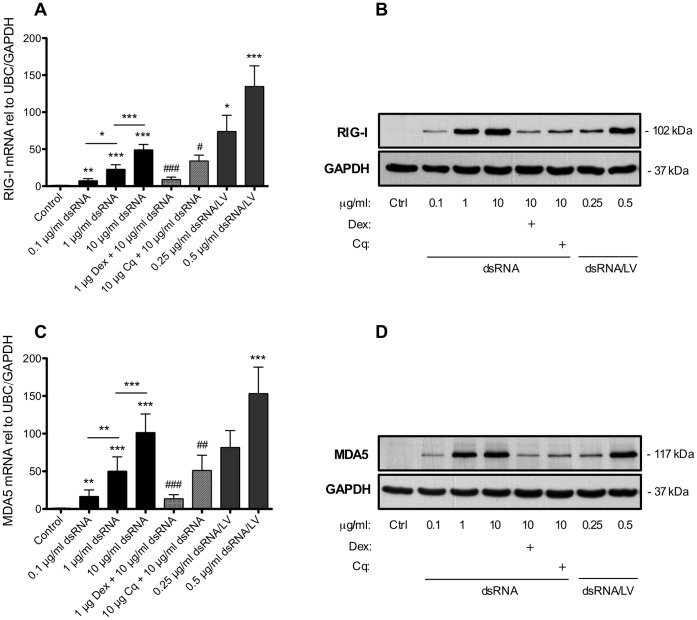
dsRNA and dsRNA/LyoVec upregulate expression of the RLRs RIG-I and MDA5 in BSMCs. BSMC were treated with dsRNA or the RLR ligand dsRNA/LyoVec (dsRNA/LV) for 24 h. mRNA (A, C) and protein (B, D) expression of RIG-I (A, B) and MDA5 (C, D) was increased concentration-dependently. n = 9 for mRNA analysis and n = 6 for protein analysis (BSMCs from three individual donors). mRNA data are presented as mean with SEM and representative western blot images of each receptor protein are shown. *p≤0.05, **p≤0.01 and ***p≤0.001 compared to non-stimulated cells (control). ^#^p≤0.05, ^##^p≤0.01 and ^###^p≤0.001 compared to 10 µg/ml dsRNA.

To verify the presence of actual RIG-I-like receptors in BSMCs we analysed RIG-I and MDA5 expression. Both RIG-I and MDA5 mRNA expression was significantly increased in a concentration-dependent manner by dsRNA treatment for 24 h (Figure 3A and 3C). The dsRNA-induced RLR expression was inhibited by dexamethasone and partly reduced by chloroquine (Figure 3A and 3C). RIG-I (Figure S2) and MDA5 protein levels were quite low but still detectable at baseline. A marked upregulation of both RIG-I and MDA5 protein expression occurred after 24 h dsRNA treatment (Figure 3B and 3D). This effect was reduced by dexamethasone and chloroquine (Figure 3B and 3D). Moreover, the RLR-agonist, dsRNA/LyoVec, also produced a pronounced effect on gene and protein expression of both RIG-I and MDA5 (Figure 3).

### dsRNA-induced BSMC Expression of the Proinflammatory Cytokines TNF-α and CXCL8

**Figure 4:**
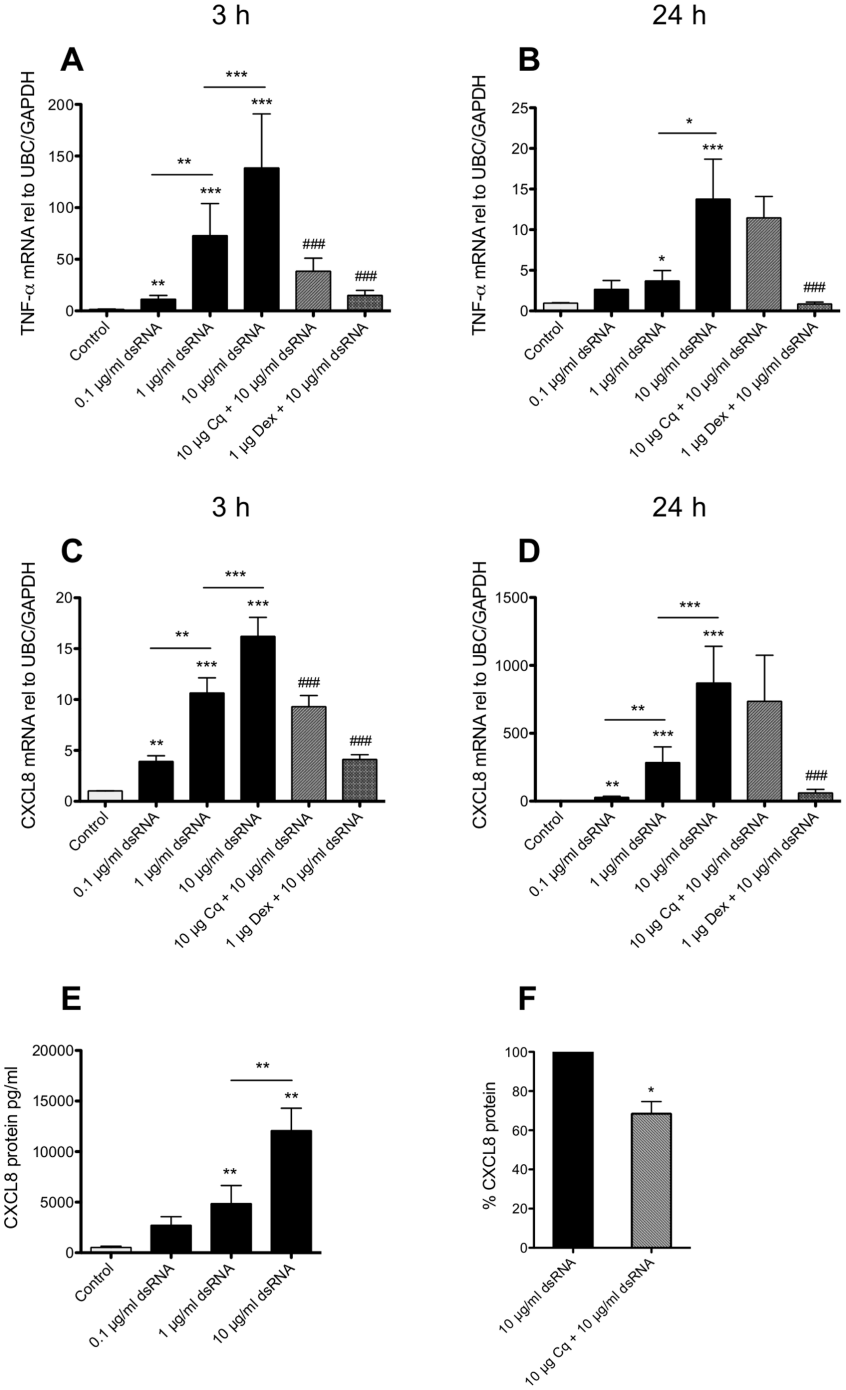
dsRNA increase expression of TNF-α and CXCL8 in BSMCs. BSMCs were stimulated with dsRNA for 3 h (A, C) and 24 h (B, D). mRNA expression of TNF-α (A-B) and CXCL8 (C-D) was increased concentration-dependently. CXCL8 protein was secreted into cell supernatants (E) and partly reduced by chloroquine (F). Data are presented as mean with SEM and n = 8–10 (BSMCs from three individual donors). *p≤0.05, **p≤0.01 and ***p≤0.001 compared to non-stimulated cells (control). ^###^p≤0.001 compared to 10 µg/ml dsRNA.

We also ascertained that dsRNA induced proinflammatory cytokine expression in our *in vitro* system. At 3 h mRNA expression of the proinflammatory cytokines TNF-α (150-fold) and CXCL8 (15-fold) was significantly and concentration-dependently increased by dsRNA (Figure 4A and 4C). At 24 h after dsRNA treatment, mRNA expression of TNF-α was less increased (15-fold), whereas the expression of CXCL8 was much further enhanced (800-fold) (Figure 4B and 4D). These data agree with previous observations demonstrating increased expression of CXCL8 and TNF-α in cultured human BSMCs after viral stimulation or IgE exposure [27], [34]. Pretreatment with dexamethasone inhibited the effects of dsRNA on TNF-α and CXCL8 after both 3 h and 24 h treatment, whereas chloroquine reduced early (3 h) mRNA expression of both cytokines (Figure 4A–D). Further, CXCL8 protein in the BSMC culture medium was substantially increased after 24 h dsRNA stimulation (Figure 4E) and this response was partly attenuated by chloroquine (Figure 4F). TNF-α protein could not be detected in cell supernatants (data not shown).

### Infection of BSMCs with RV1B Induces Expression of IFNs and RLRs

**Figure 5:**
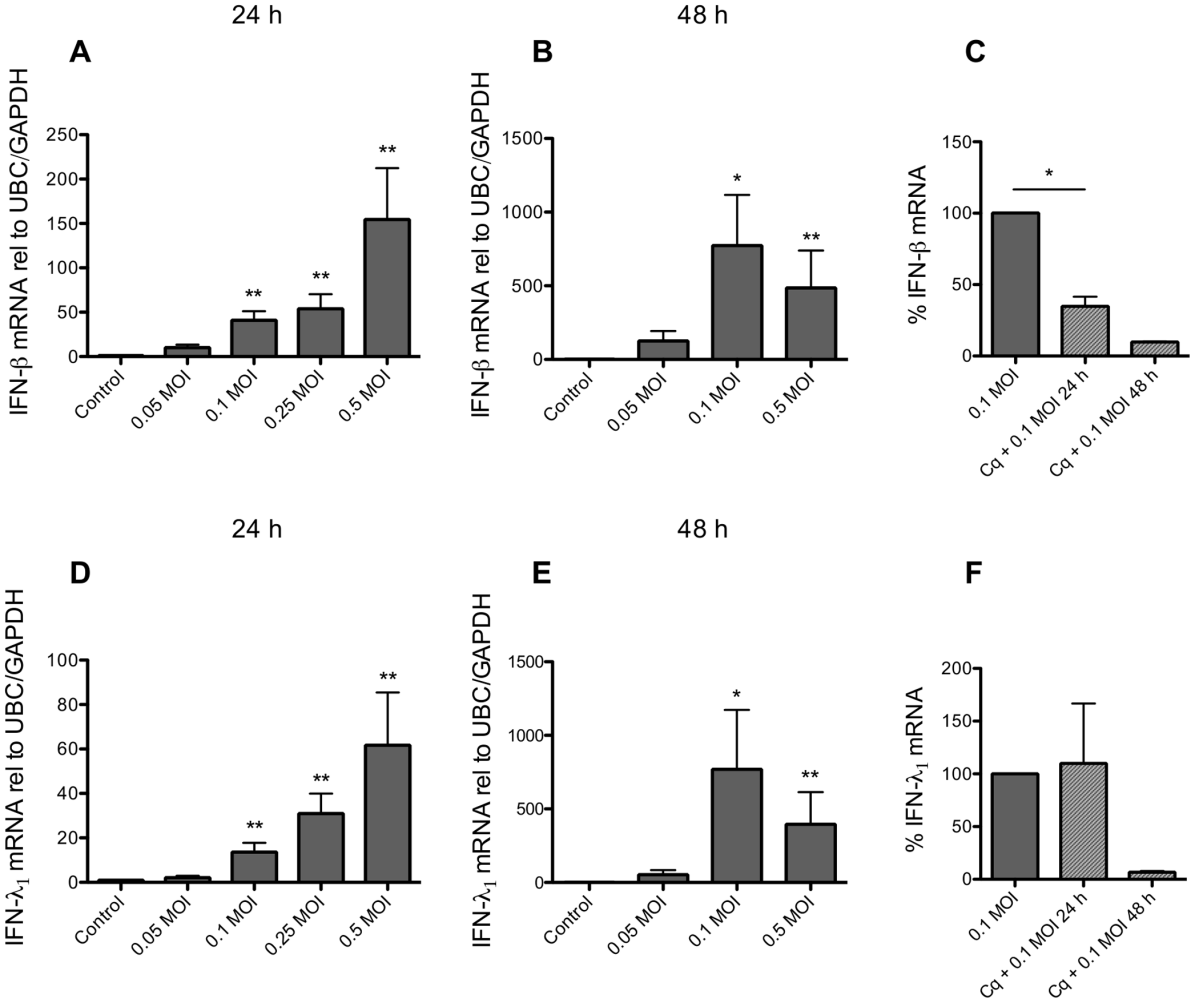
RV1B induces BSMC mRNA expression of IFNs. BSMCs exposed to RV1B at increasing doses (MOI = multiplicity of infection) induced expression of IFN-β (A) and IFN-λ_1_ (D) 24 h post-infection as determined by RT-qPCR. IFN-β and IFN-λ_1_ expression was further enhanced after 48 h (B, E). Chloroquine (10 µg/ml) reduced RV1B-induced IFN-β expression after 24 and 48 h (C), whereas IFN- λ_1_ expression was reduced after 48 h only (F). Data are presented as mean with SEM and n = 6 for 24 h RV1B infection, n = 3 for 48 h infection and n = 2–3 for chloroquine experiments (BSMCs from three individual donors). *p≤0.05 and **p≤0.01 compared to non-infected cells (control).

**Figure 6:**
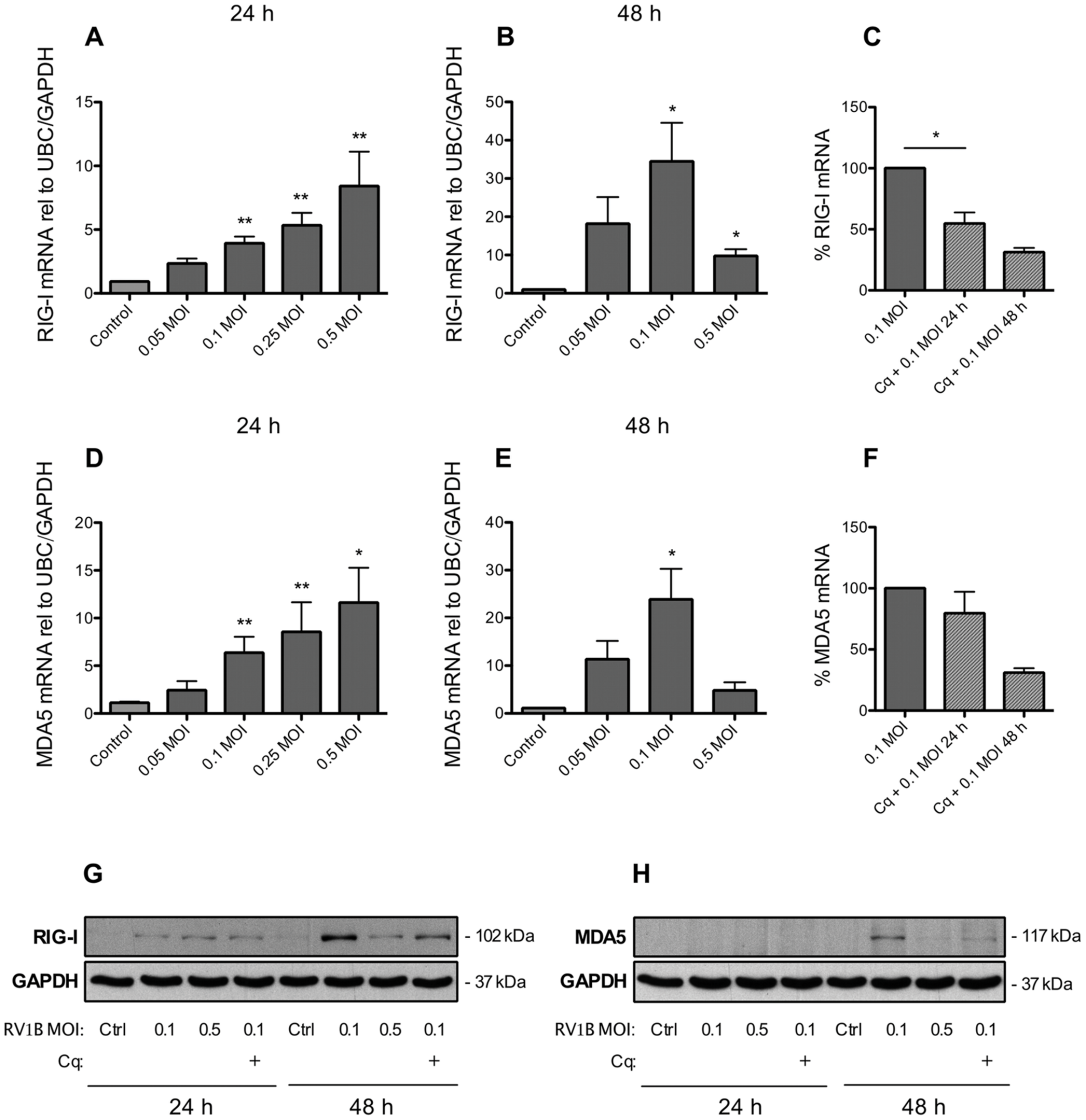
BSMC expression of RIG-I and MDA5 is upregulated upon RV1B infection. Infection with increasing doses (MOI) of RV1B for 24 h upregulated BSMC mRNA expression of RIG-I (A) and MDA5 (D) and the expression of both RLRs were further enhanced 48 h post-infection (B, E). Chloroquine (10 µg/ml) partly reduced the effect of RV1B on both RIG-I and MDA5 mRNA expression, especially after 48 h (C, F). RIG-I protein (G) was upregulated at 24 h and more enhanced at the infection dose of 0.1 MOI 48 h post-infection. MDA5 protein (H) was upregulated 48 h post-infection only. Chloroquine reduced RV1B-induced effects on both RIG-I (G) and MDA5 (H) protein at 48 h. Data are presented as mean with SEM and n = 6 for 24 h RV1B infection, n = 3 for 48 h infection and n = 2–3 for chloroquine experiments (BSMCs from three individual donors). Representative western blot images of each receptor protein are shown. *p≤0.05 and **p≤0.01 compared to non-infected cells (control).

Separate experiments were performed to validate if rhinovirus infection produced dsRNA-like effects on IFN and RLR expression. RV1B infection for 24 h increased BSMC expression of both IFN-β (150-fold) and IFN-λ_1_ (60-fold) mRNA (Figure 5A and 5D) as well as upregulated the expression of RIG-I (10-fold) and MDA5 (12-fold) (Figure 6A and 6D). Further, similar to the effects of dsRNA, infecting BSMCs with RV1B resulted in dose-dependent expression of CXCL8 mRNA whereas TNF-α expression was not appreciably affected after 24 h viral infection (Figure S3). Since cell responses to RV infection may take longer to develop than responses to dsRNA stimuli we also infected BSMCs for 48 h. We found that mRNA levels of IFNs were further enhanced at 48 h (Figure 5B and 5E). Also mRNA of RIG-I and MDA5 was further upregulated at 48 h compared to 24 h (6B and 6E). Chloroquine reduced IFN-β mRNA at 24 h and more completely by 48 h (Figure 5C) whereas IFN-λ_1_ was only inhibited by chloroquine at 48 h. However, chloroquine also inhibited viral replication at 24 and 48 h (Figure S4). RIG-I protein expression was induced by RVIB at 24 h but much more after 48 h and was reduced by chloroquine (Figure 6G). In some contrast, MDA5 protein was not induced by RV1B at 24 h post-infection but was induced at 48 h (at the virus dose 0.1 MOI) and also reduced by chloroquine. IFN-β and IFN-λ_1_ protein in cell supernatants were only just above detection level post-infection at 24 and 48 h (data not shown).

## Discussion

This study demonstrates for the first time that dsRNA, a mimic of viral infection and a potential danger signal derived from damaged or dead cells, induces cytosolic RLRs as well as production of IFN-β and IFN-λ_1_ in human primary BSMCs. Hence, although the early dsRNA-induced expression of the IFNs was dependent on endosomal TLR3 activation, TLR3 was not the only responsive receptor in this study. We found that the RLRs, RIG-I and MDA5, were clearly upregulated at both mRNA and protein level in BSMCs by dsRNA and RV infection. Further, as suggested by pronounced effects of a specific agonist, the cytosolic RLRs have a capacity to be functionally involved in BSMC production of IFN-β and IFN-λ_1_ proteins. The present data highlight the possibility that cytosolic RLRs jointly with TLR3 in BSMCs contribute to airway antiviral defence and inflammatory responses.

The presence of RV in bronchial subepithelial mucosa layer [3] suggests that RV may have important effects also on bronchial fibroblasts and smooth muscle cells. Exposure to RV also results in fibroblast and BSMC production of proinflammatory cytokines [27], [31]. However, findings in human cultured airway fibroblasts indicate that this cell type does not contribute significantly to RV-induced antiviral responses [31]. RV infection failed to increase IFN-β mRNA and protein production in fibroblasts derived from bronchial biopsies from asthmatic and healthy donors [31]. RV infection also failed to produce IFN-λ_1_ in parenchymal fibroblasts derived from healthy human lungs [35]. Interestingly, it was suggested that these bronchial structural cells could serve as an important RV replication reservoir [31]. This latter aspect would increase the possibility of infection also of the BSMCs, applying especially to asthmatic bronchi where the smooth muscle component is increased and where smooth muscle cells may grow close to the epithelial lining [36], [37]. Hence, the present demonstration of BSMC generation of IFNs suggests that these cells may contribute to host defence in bronchial RV infections.

Viral infected cells undergo lysis spreading the infection locally. However, in asthmatic bronchi and especially in severe stages of the disease lysis or necrosis of different cell types, including neutrophils [38], eosinophils [39], and possibly smooth muscle cells [9], too, occur also independent of infection. Released necrosis-derived self-RNA molecules or secondary structure may induce significant danger responses [10], [11], [12], [13]. These responses appear to be well mimicked by dsRNA as demonstrated in mouse models [10], and human esophageal epithelial cells [11]. Hence, the present findings of dsRNA-induced BSMC responses suggest the possibility that BSMCs contribute to RLR- as well as TLR3-mediated antiviral and proinflammatory proteins in severe bronchial disease conditions where necrotic cells abound. It is of note that dexamethasone, representing the mainstay treatment of severe asthma, inhibited not only dsRNA-induced BSMC generation of proinflammatory cytokines (TNF-α and CXCL8) but also IFN-β and IFN-λ_1_ in this study. Hence, this important class of anti-asthma drugs would not spare the contribution of BSMCs to antiviral innate immunity.

Our finding that BSMCs have a capacity to produce IFN-λ_1_ in response to dsRNA stimuli may have dual implications. First, IFN-λ_1_ may serve as a mediator in host defence as IFN-λs, similar to type I IFNs, exert direct inhibitory actions on viral replication and activate the apoptotic machinery in viral-infected cells [40]. Second, BSMC production of IFN-λ_1_ may also be causative in pathogenic events in the respiratory tract as suggested by a recent comprehensive study in asthmatic children where RV-induced exacerbations were exclusively associated with increased levels of IFN-λ_1_ [21]. However, the mechanisms behind this finding and the role of asthmatic BSMCs in IFN-λ_1_ production need further investigation [23].

A previous study reported that different strains of Chlamydia induced the production of low levels of IFN-β in BSMCs when infected cells were treated with TNF-α [41]. However, the authors did not examine PRR involvement in this response. Using neutralizing antibodies to IFN-β Tliba *et al* further observed that autocrine IFN-β is involved in regulation of BSMC expression of cytokines by TNF-α [42]. Since the dsRNA-induced expression of TNF-α mRNA in this study was not associated with detectable levels of TNF-α protein, autocrine effects of TNF-α may not be involved in the present prompt production of IFN-β by BSMCs. The early (3 h) dsRNA-induced increase in IFN-β mRNA in the present study was inhibited by chloroquine that is known to inhibit activation of endosomal TLR3 [30]. Hence, we cannot exclude the possibility that an initial TLR3-mediated production of IFN-β has contributed through its known capacity to amplify IFN production in an autocrine or paracrine manner [43]. Chloroquine reduced the effects of RV1B infection but it also reduced RV1B replication suggesting that chloroquine-mediated effects on IFNs may be infection-dependent rather than solely TLR3-dependent. Importantly, chloroquine did not reduce the stimulant effects of dsRNA/LyoVec on BSMC expression of IFNs. This latter observation agrees with our previous data obtained in human bronchial epithelial cells [33] and support the view that dsRNA/LyoVec acts on RLRs rather than on TLR3.

Discussion of the present data is somewhat hampered by lack of previous BSMC data on RLRs and the fact that relative roles of TLR3, MDA5 or RIG-I in viral infections are cell-type and virus specific [4], [5], [44], [45]. Yet, it is of interest to compare the present findings with previous observations involving human bronchial epithelial cells where the roles of these three PRRs in viral induced IFNs have been examined in some detail [4], [5]. Wang *et al* [4] and Slater *et al* [5] demonstrated that RV infection increases epithelial cell expression of IFN-β and IFN-λ_1_ mRNA (neither Wang *et al* [4] nor Slater *et al* [5] reported data on production of IFN proteins). Using BEAS-2B cells and siRNA transfection Wang *et al* further reported that MDA5 and TLR3 (using siRNA against the TLR3 adaptor TRIF) but not RIG-I were important for RV-induced gene expression of IFN-β and IFN-λ_1_ [4]. Using similar siRNA methodology in human primary bronchial epithelial cells Slater and colleagues found a partly different regulatory scheme: TLR3 and MDA5 were required for IFN-λ_1_ whereas TLR3, MDA5, and RIG-I were required for RV-induced IFN-β mRNA expression [5]. Slater *et al* [5] suggest the possibility that the differences in role of RIG-I may reflect differences between the immortalized cell line, BEAS-2B used by Wang *et al* [4], and the primary bronchial cells they used. They further discuss possible drawbacks with the siRNA approach including inefficient knockout of the specific target mRNA and unspecific off-target interference with endogenous RNA-sensing molecules. However, they conclude that compelling evidence support their view that both RIG-I and MDA5 are important in innate responses to RV infection [5]. This conclusion may also apply to primary BSMCs with regard to the consistent effect on RIG-I as well as MDA5 that were induced in the present study along with IFNs in response to RV and dsRNA with particularly pronounced increase in IFN-β and IFN-λ_1_ production induced by the cytosolic RLR stimulant, dsRNA/LyoVec. Slater *et al* further provided evidence that initial TLR3 activation contributed to inducement of RIG-I and MDA5 [5]. This possibility is of interest for further study in BSMCs since we observed that the early response to dsRNA involved TLR3 and that both RIG-I and MDA5 were then markedly induced and associated with more sustained, and likely more important BSMC production of IFN-β and IFN-λ_1_. Future studies are required to delineate details of the interaction between TLR3 and RLRs in rhinovirus- and dsRNA-exposed BSMCs.

To summarise, in this study we provide evidence that support a potential role of BSMCs in innate immune responses to respiratory viral infections and to necrotic cells. Our results thus demonstrate for the first time that rhinovirus (RV1B) induces, from low baseline levels, both RIG-I and MDA5 as well as type I and type III IFN mRNA in BSMCs. As demonstrated with dsRNA stimuli, and especially with the cytosolic stimulant dsRNA/LyoVec, the potential contribution of the RLRs to increased expression and production of IFNs by BSMCs is significant. As suggested by effects of chloroquine, known to inhibit endosomal acidification, TLR3 could be responsible for initial dsRNA-induced upregulation of RIG-I and MDA5. In conclusion, we believe that our findings have provided new insight into how BSMCs may be involved in modulation of inflammatory and innate immune responses during respiratory viral infections and potentially in severe bronchial disease presenting necrotic cells in the bronchial wall.

## Supporting Information

Figure S1 **dsRNA/LV induces BSMC expression of IFNs independent of endosomal TLR3 activation.** Chloroquine (10 µg/ml) treatment 1 h prior to stimulation with dsRNA/LV did not inhibit dsRNA/LV-induced mRNA expression of IFN-β (A, B) and IFN-λ_1_ (C, D) at neither 3 h nor 24 h. Data are presented as mean with SEM and n = 4 (BSMCs from three individual donors). *p≤0.05 and **p≤0.01 compared to non-stimulated cells (control).
(TIF)

Figure S2 **RIG-I protein is detectible at baseline in BSMCs.** Representative western blot image demonstrating the presence of RIG-I protein in non-treated (control) BSMCs.
(TIF)

Figure S3 **RV1B triggers expression of proinflammatory cytokines in BSMCs.** TNF-α mRNA expression (A, B) in BSMCs was mainly induced by RV1B after 48 h, whereas expression of CXCL8 was increased at both 24 h (D) and 48 h (E) post-infection. The effect of RV1B on 48 h proinflammatory mRNA expression was reduced by chloroquine (10 µg/ml) (C, F). Data are presented as mean with SEM and n = 6 for 24 h RV1B infection, n = 3 for 48 h infection and n = 2–3 for chloroquine experiments (BSMCs from three individual donors). *p≤0.05 and **p≤0.01 compared to non-infected cells (control).
(TIF)

Figure S4 **Time- and dose-dependent RV1B replication in BSMCs is partly inhibited by chloroquine.** BSMCs infected with RV1B (0.1 and 0.5 MOI) for 24 and 48 h (A) showed a time- and dose-dependent expression of intracellular viral RNA (vRNA) as determined by RT-qPCR. Chloroquine (10 µg/ml) partly inhibited RV1B replication after both 24 and 48 h (B). Data are presented as mean with SEM and n = 3–5 for RV1B infection only and n = 2–3 for chloroquine experiments (BSMCs from three individual donors). *p≤0.05 and **p≤0.01.
(TIF)
