# Supplementary figures and images for: Rhinovirus and dsRNA Induce RIG-I-Like Receptors and Expression of Interferon β and λ1 in Human Bronchial Smooth Muscle Cells
Source: PLoS One. 2013 Apr 29;8(4):e62718. doi: 10.1371/journal.pone.0062718 (PMC3639170; doi:10.1371/journal.pone.0062718)

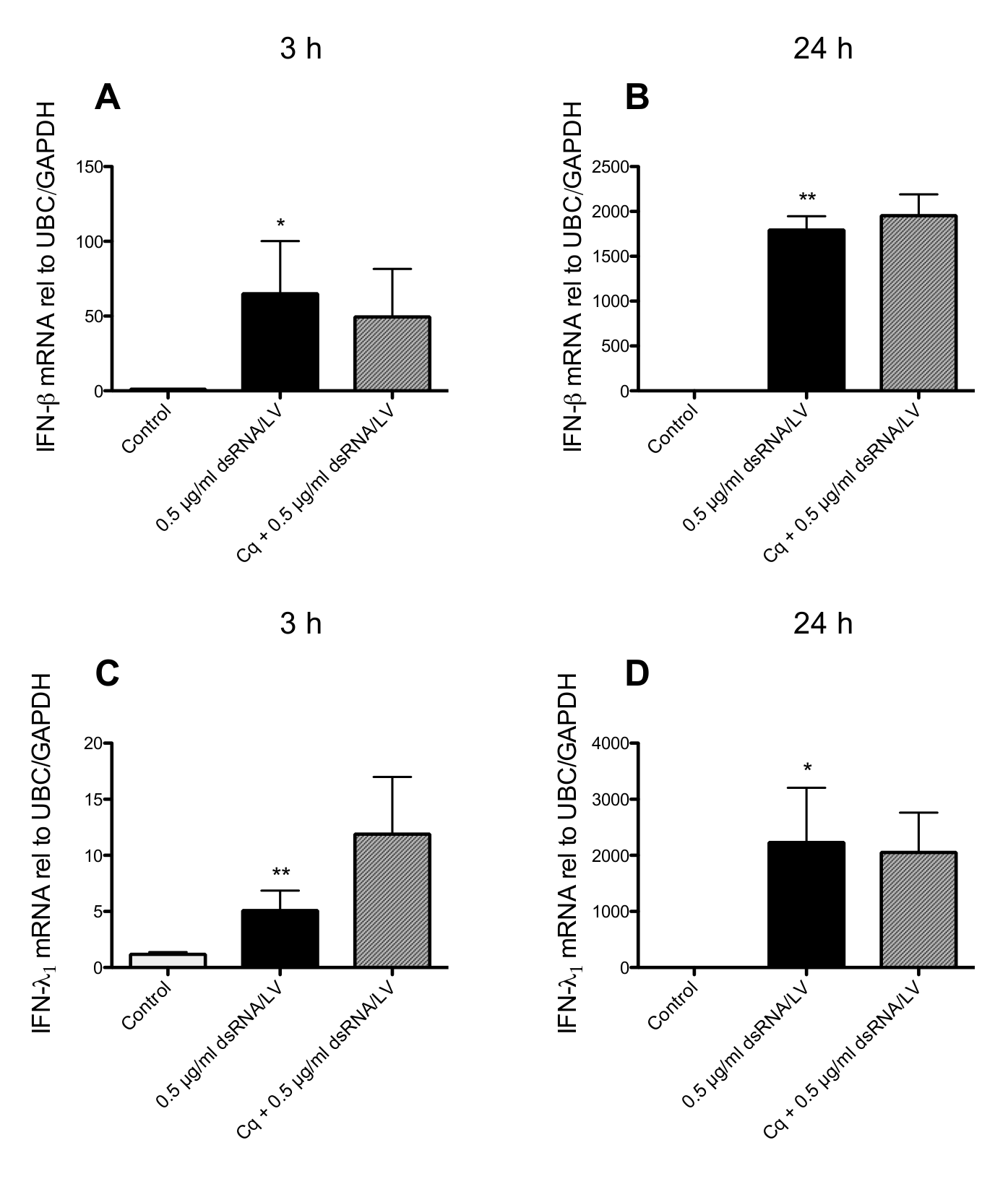

Supplement: Figure S1 — dsRNA/LV induces BSMC expression of IFNs independent of endosomal TLR3 activation. Chloroquine (10 µg/ml) treatment 1 h prior to stimulation with dsRNA/LV did not inhibit dsRNA/LV-induced mRNA expression of IFN-β (A, B) and IFN-λ1 (C, D) at neither 3 h nor 24 h. Data are presented as mean with SEM and n = 4 (BSMCs from three individual donors). *p≤0.05 and **p≤0.01 compared to non-stimulated cells (control). (TIF) [file pone.0062718.s001.tif]

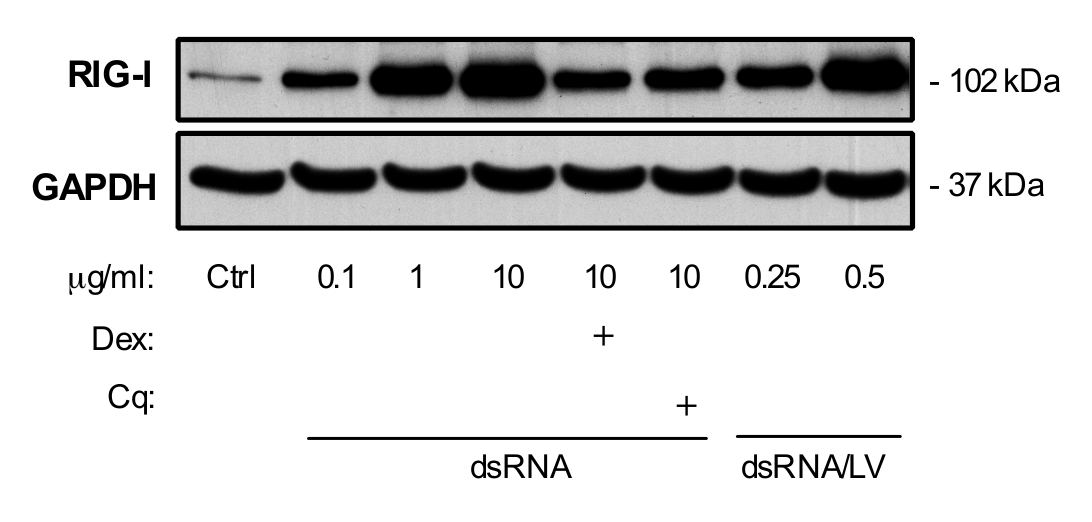

Supplement: Figure S2 — RIG-I protein is detectible at baseline in BSMCs. Representative western blot image demonstrating the presence of RIG-I protein in non-treated (control) BSMCs. (TIF) [file pone.0062718.s002.tif]

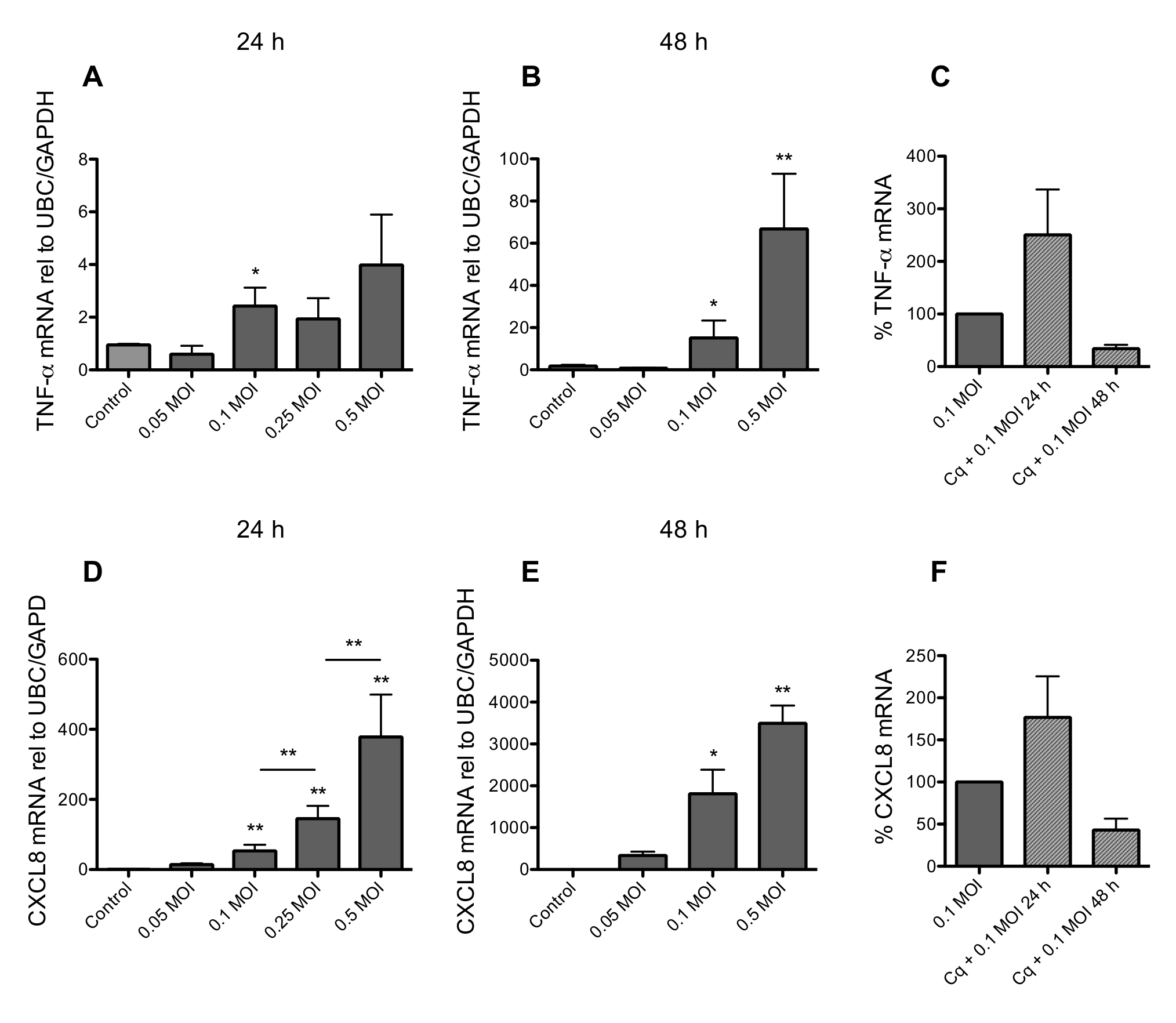

Supplement: Figure S3 — RV1B triggers expression of proinflammatory cytokines in BSMCs. TNF-α mRNA expression (A, B) in BSMCs was mainly induced by RV1B after 48 h, whereas expression of CXCL8 was increased at both 24 h (D) and 48 h (E) post-infection. The effect of RV1B on 48 h proinflammatory mRNA expression was reduced by chloroquine (10 µg/ml) (C, F). Data are presented as mean with SEM and n = 6 for 24 h RV1B infection, n = 3 for 48 h infection and n = 2–3 for chloroquine experiments (BSMCs from three individual donors). *p≤0.05 and **p≤0.01 compared to non-infected cells (control). (TIF) [file pone.0062718.s003.tif]

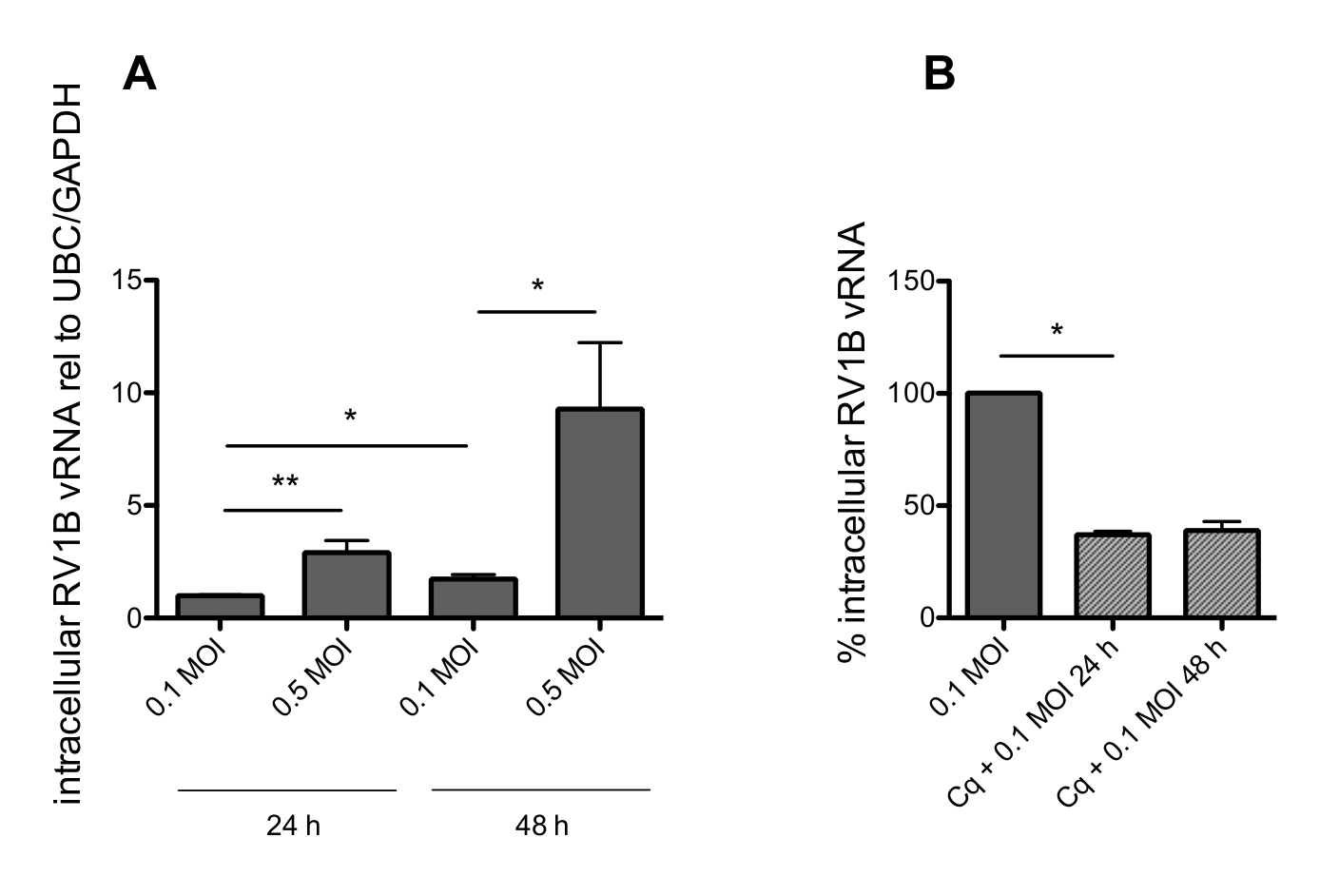

Supplement: Figure S4 — Time- and dose-dependent RV1B replication in BSMCs is partly inhibited by chloroquine. BSMCs infected with RV1B (0.1 and 0.5 MOI) for 24 and 48 h (A) showed a time- and dose-dependent expression of intracellular viral RNA (vRNA) as determined by RT-qPCR. Chloroquine (10 µg/ml) partly inhibited RV1B replication after both 24 and 48 h (B). Data are presented as mean with SEM and n = 3–5 for RV1B infection only and n = 2–3 for chloroquine experiments (BSMCs from three individual donors). *p≤0.05 and **p≤0.01. (TIF) [file pone.0062718.s004.tif]
